# Machine learning algorithms for detection of visuomotor neural control differences in individuals with PASC and ME

**DOI:** 10.3389/fnhum.2024.1359162

**Published:** 2024-04-04

**Authors:** Harit Ahuja, Smriti Badhwar, Heather Edgell, Marin Litoiu, Lauren E. Sergio

**Affiliations:** ^1^School of Information Technology, York University, Toronto, ON, Canada; ^2^School of Kinesiology and Health Science, York University, Toronto, ON, Canada; ^3^Lassonde School of Engineering, York University, Toronto, ON, Canada

**Keywords:** PASC, machine learning, Myalgic Encephalomyelitis/chronic fatigue syndrome (ME/CFS), Cognitive-Motor Integration, synthetic data

## Abstract

The COVID-19 pandemic has affected millions worldwide, giving rise to long-term symptoms known as post-acute sequelae of SARS-CoV-2 (PASC) infection, colloquially referred to as long COVID. With an increasing number of people experiencing these symptoms, early intervention is crucial. In this study, we introduce a novel method to detect the likelihood of PASC or Myalgic Encephalomyelitis (ME) using a wearable four-channel headband that collects Electroencephalogram (EEG) data. The raw EEG signals are processed using Continuous Wavelet Transform (CWT) to form a spectrogram-like matrix, which serves as input for various machine learning and deep learning models. We employ models such as CONVLSTM (Convolutional Long Short-Term Memory), CNN-LSTM, and Bi-LSTM (Bidirectional Long short-term memory). Additionally, we test the dataset on traditional machine learning models for comparative analysis. Our results show that the best-performing model, CNN-LSTM, achieved an accuracy of 83%. In addition to the original spectrogram data, we generated synthetic spectrograms using Wasserstein Generative Adversarial Networks (WGANs) to augment our dataset. These synthetic spectrograms contributed to the training phase, addressing challenges such as limited data volume and patient privacy. Impressively, the model trained on synthetic data achieved an average accuracy of 93%, significantly outperforming the original model. These results demonstrate the feasibility and effectiveness of our proposed method in detecting the effects of PASC and ME, paving the way for early identification and management of the condition. The proposed approach holds significant potential for various practical applications, particularly in the clinical domain. It can be utilized for evaluating the current condition of individuals with PASC or ME, and monitoring the recovery process of those with PASC, or the efficacy of any interventions in the PASC and ME populations. By implementing this technique, healthcare professionals can facilitate more effective management of chronic PASC or ME effects, ensuring timely intervention and improving the quality of life for those experiencing these conditions.

## 1 Introduction

The COVID-19 pandemic had an unparalleled effect on global health, causing widespread morbidity and mortality. In addition to the acute phase of the illness, a considerable number of individuals encounter long-term symptoms following recovery from the initial stage (Di Toro et al., 2021). Termed post-acute sequelae of SARS-CoV-2 infection (PASC) or, colloquially, “long COVID” (Raveendran et al., 2021), there is concern that these individuals will go on to experience Myalgic Encephalomyelitis (ME), commonly referred to as chronic fatigue syndrome. ME is a chronic, often debilitating condition characterized by severe fatigue, neurological issues, and a range of flu-like symptoms that are not alleviated by rest. The etiology of ME remains unclear, although it commonly occurs after viral infections, suggesting a post-viral syndrome potentially linked to disruptions in immune system functioning. ME has consistently presented a complex challenge for patients and healthcare providers, with its array of symptoms often leading to significant delays in diagnosis and effective management (Ghali et al., 2022). In examining the aftermath of viral epidemics, contemporary research provides insight into the long-term consequences experienced by patients. A recent study analyzed a sample of 465 patients with PASC and found that 58% met the criteria for ME, indicating substantial occurrences of post-viral conditions after the COVID-19 health crisis (Jason and Dorri, 2022). This trend is particularly concerning, given the parallels between the symptom profiles of ME and PASC. This concern is reinforced by multiple studies highlighting the long-term health challenges following COVID-19 and other viral outbreaks (Herridge et al., 2003; Moldofsky and Patcai, 2011; Komaroff and Bateman, 2021; Logue et al., 2021; Movahed and Rezaeian, 2022). Given these insights, the importance of early identification becomes even more pronounced, highlighting the need for timely interventions to manage these long-term effects efficiently. These commonalities include fatiguability, sleep abnormalities, musculoskeletal pain, weakness, memory loss, impaired eye-hand coordination, and brain fog. Collectively, these symptoms present numerous challenges to the healthcare industry specifically and the household and labor force more generally, given the over nine billion dollars U.S. in estimated productivity loss from ME patients alone (Reynolds et al., 2004).

The pandemic has accelerated the adoption of various technologies in healthcare, including the Internet of Things (IoT). IoT is a term that encapsulates the process of integrating everyday objects into the digital sphere through internet connectivity, thereby enabling these objects to transmit and receive data. Advancements in IoT technology have played a crucial role in neuroscientific research by enabling the collection of critical health data from wearable devices, thus making health monitoring more accessible and user-friendly (Pap et al., 2020). Specifically, scientists have been utilizing Electroencephalogram (EEG) signals collected with IoT devices to gain insights into and identify these cognitive impairments. This non-invasive technique captures the brain's electrical activity and has shown promise in predicting neurological disorders (Alturki and AlSharabi, 2020).

However, one of the challenges in this context is the limited availability and accessibility of user-friendly, wearable EEG devices for widespread monitoring and indentification of PASC and ME symptoms. To address this challenge, we introduce a novel method to detect the likelihood of individuals developing such chronic conditions using a commercially available four-channel headband that collects EEG data. Exploring cognitive load and working memory capacity, concepts previously investigated in e-learning (Xiong and Kong, 2020), can provide critical insights into these cognitive impairments.

Machine Learning (ML), an application of artificial intelligence, offers powerful tools for identifying patterns and making predictions from complex datasets, particularly useful in analyzing EEG signals. In studying cognitive impairments, machine learning's capacity to discern subtle patterns in brain activity data, often overlooked by human inspection alone, can have significant implications for early and accurate diagnosis. Recent studies have effectively used machine learning methods and EEG data in various ways. One research paper explored the impact of high altitude on attention control processes, using machine learning to identify cases of chronic high-altitude hypoxia (Liu et al., 2022). In another study, a blend of Convolutional Neural Networks (CNN) and machine learning classifiers were applied, achieving impressive results in detecting epilepsy with high accuracy across EEG datasets (Hassan et al., 2022). These examples highlight the wide range of uses and potential benefits of machine learning alongside EEG data for detecting cognitive impairments. This approach could be extended to cognitive disorders, including PASC and ME, to facilitate early and accurate diagnosis.

Another significant challenge is the need for a robust and standardized methodology to compare the performance of various machine learning and deep learning models for PASC/ME effects detection using EEG data. Our study addresses this issue by transforming raw EEG signals via Continuous Wavelet Transform (CWT) into a spectrogram-like matrix (Chaudhary et al., 2021). This matrix is then applied to train diverse models, including CONVLSTM (Convolutional Long Short-Term Memory; Shi et al., 2015), CNN-LSTM (Hochreiter and Schmidhuber, 1997; Krizhevsky et al., 2012), and Bi-LSTM (Bidirectional Long short-term memory; Graves et al., 2013). These models are trained and are compared with traditional machine learning models in our analysis. As per our preliminary results, the CNN-LSTM model demonstrated the highest efficacy, showing substantial promise in distinguishing between healthy participants and those affected by PASC or ME.

A distinct aspect of our approach involves generating synthetic data to enhance the machine learning models used in our study. Given the challenges in obtaining large volumes of EEG data and respecting patient privacy, we employ Wasserstein Generative Adversarial Networks (WGANs; Arjovsky et al., 2017) to generate synthetic spectrograms. These synthetic spectrograms mimic the EEG patterns in healthy individuals and those suffering from PASC and ME. By using WGANs, we can significantly augment our dataset without compromising patient confidentiality. The synthetic spectrograms are processed separately for healthy and PASC + ME participants to maintain distinct data classes, allowing for focused analysis. Incorporating this synthetic data into the training phase has shown promising results, notably improving the model's performance metrics. Therefore, synthetic data addresses critical challenges such as data volume and privacy and shows significant potential for improving the model's predictive capabilities. On a related note, recent studies highlight the value of synthetic data for safeguarding privacy, particularly in healthcare applications. By applying GANs (Goodfellow et al., 2014), one such study proved that synthetic medical records are not just high-quality but also offer formidable protection against potential data leaks (Ashrafi et al., 2023). In this study, we describe the implementation of machine learning and deep learning models to distinguish brain wave activity collected from individuals affected by PASC or ME from healthy control participants during the performance of a challenging rule-based visuomotor skill task.

The remainder of this paper is organized as follows: Section 2 describes the methodology used in our study, including data collection, preprocessing, and the implementation of machine learning and deep learning models. Section 3 presents the evaluation and results of our experiments. Finally, Section 4 concludes the paper and outlines directions for future research.

## 2 Data generation and preprocessing

### 2.1 Overview

**Figure 1:**
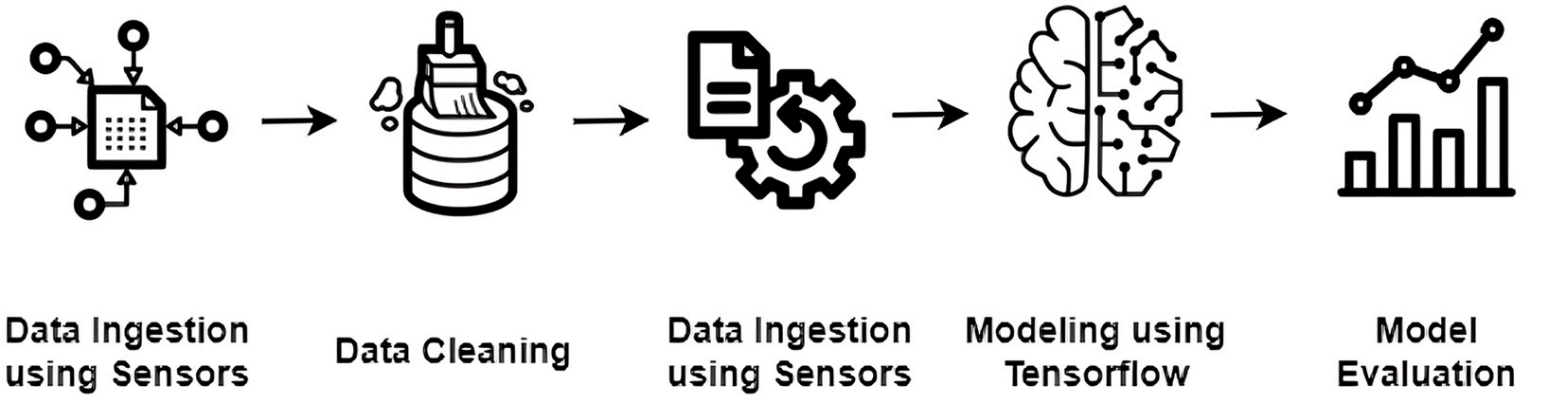
Sequential methodology for EEG data processing and ML model development in PASC and ME detection.

This section outlines our comprehensive process for generating and preparing data, focusing on the detailed collection, cleaning, and augmentation of data derived from a Cognitive-Motor Integration (CMI) task. Our aim is to enhance the data's quality and reliability to ensure it is well-prepared for analysis. Figure 1 illustrates the step-by-step methodology for processing EEG data tailored for detecting effects of PASC and ME.

### 2.2 Participants

The behavioral data and electroencephalogram recordings were collected from 23 participants, as detailed in Table 1. This group included 10 healthy control participants, six participants with PASC, and seven participants with ME. The healthy participants had no history of brain injury or neurological illness. All procedures were approved by York University's Human Participants Research Committee, and all participants provided informed consent to participate.

**Table 1:** Distribution of participants by group, average age (mean, std. dev.), and health condition.

**Group**	**Number of participants**	**Age (mean)**	**Age (Std. dev.)**
Control	10	31.6	18.24
ME	7	45.71	10.33
PASC	6	44.67	18.45
ME/PASC combined	13	45.23	14.57

### 2.3 Experimental task

**Figure 2:**
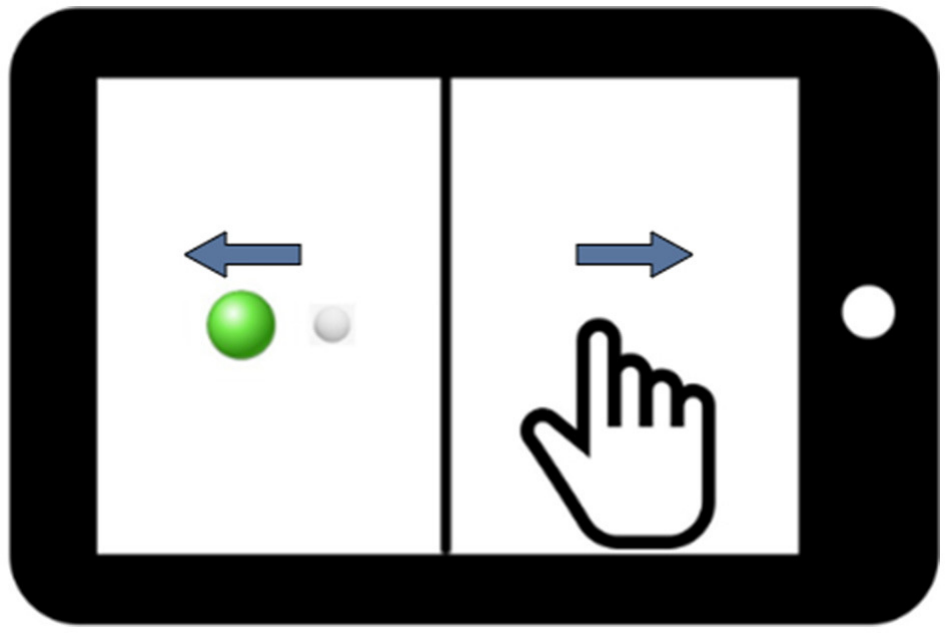
An illustration of the tablet screen divided into two halves by a vertical line. The white dot represents the cursor that the participant controls, and the green dot denotes the target. Blue arrows display the visual feedback reversal, which requires Cognitive-Motor Integration for successful task completion in addition to the spatial decoupling of hand and target.

In this study, we employed a between-subjects design. This design involved using two distinct groups of participants to compare the effect of health on performance. The between-subjects design is advantageous in preventing potential carry-over or learning effects that may influence the results when a single participant is exposed to multiple conditions. In our case, the two groups comprised healthy participants and the combined PASC and ME participants. The PASC and ME groups were combined based on their kinematic behavioral performance not being significantly different. Participants performed computer-based visuomotor skill evaluation tasks using the BrDI application (BrDI: Brain Dynamics Indicator, 3MotionAI Inc.). They sat at a desk with a 10.1-inch tablet (Samsung Galaxy Tab) within easy reach for these tasks. The task, as depicted in Figure 2, involved using the index finger of the participant's dominant hand to navigate a cursor (white dot, 5 mm diameter) from the screen to one of the four peripheral targets (up, down, left, or right relative to center).

Beginning the trial required guiding the cursor to a solid green circle (Figure 2). After holding the cursor there for 2 s, a green circle appeared at one of the peripheral targets. The trial concluded when the cursor remained in the final target for ~500 ms. The task had 20 trials: five trials directed toward each of the four targets. The task demanded the integration of spatial and cognitive rules, requiring participants to perform a more complex task involving CMI relative to directly moving the finger to a target. Specifically, on the tablet, a vertical line split the display such that the location of the required hand movement and the guiding visual information were spatially decoupled. In addition, the cursor feedback was inverted by 180 degrees, requiring participants to slide their finger in one direction to move the cursor in the opposite direction to reach the target. Participants were instructed to move as quickly and accurately as possible and were allowed two practice trials to ensure they understood the task. They were further instructed to minimize unnecessary eye blinking and jaw clenching, as these actions could introduce noise into the EEG data and potentially affect the study's results. The task took ~5 min to complete. Previous findings from our groups have reliably demonstrated the sensitivity of this task to detect brain network dysfunction during the control of rule-based visuomotor skill performance (Salek et al., 2011; Hawkins and Sergio, 2014; Hawkins et al., 2015; Hurtubise et al., 2016, 2020; Sergio et al., 2020; Smeha et al., 2022).

### 2.4 Data acquisition and recording

**Figure 3:**
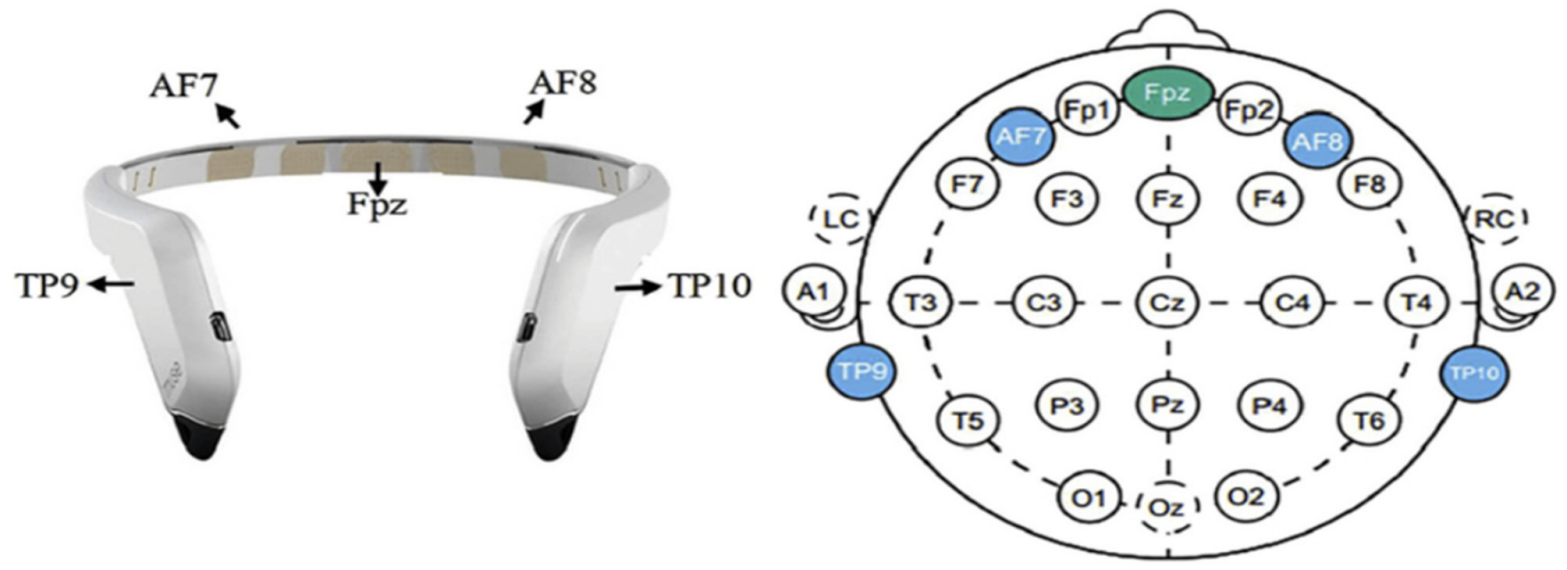
Muse 2 headband and the 10–20 system of electrode placement for Muse.

In this study, EEG data collection was critical to our analysis. We utilized the portable Muse2^TM^ headband system (InteraXon Inc., Toronto, Canada) equipped with four electrodes—TP9, TP10, AF7, and AF8—to record real-time neural activity while participants engaged in visuomotor tasks. Adhering to the International 10–20 System for electrode placement, as illustrated in Figure 3, ensured standardized and optimal positioning of electrodes for EEG data capture. The recording frequency was set at 256 Hz, optimizing the resolution of neural signal detection during the tasks. Each participant's EEG data were collected for a precise duration of ~30 s, carefully timed to encompass the entirety of the experimental task engagement, resulting in around 20,000 data points per participant.

At this initial stage of data collection, our methodology was designed to ensure a robust capture of neural dynamics across the multiple channels provided by the Muse2 system. This approach allowed us to secure a comprehensive dataset, ready for the subsequent stages of cleaning and preprocessing as outlined in Section 2.5. Emphasizing the maintenance of the raw EEG signals' fidelity, including the distinct contributions from each of the four electrodes, laid the groundwork for an in-depth examination of the complex patterns within the collected neural data.

### 2.5 Data cleaning and preprocessing

The data cleaning and preprocessing process involved a series of steps to develop a high-quality dataset appropriate for machine learning evaluation. Owing to the high sensitivity of the Muse 2 device, the EEG signals were subjected to noise interference, including disruptions caused by participant's eyes blinking or jaw clenching. Such instances introduced noise into the dataset and potentially resulted in blank values. Hence, noise elimination was an imperative step.

This was accomplished through the use of two Python libraries, NumPy and Pandas. NumPy is a powerful library that enables efficient manipulation of large numerical data arrays. Pandas, built on top of NumPy, provides tools for data cleaning, such as handling missing data. This process was critical in enhancing the dataset's efficiency by eliminating duplicate values and minimizing model bias. After cleaning the dataset, it was partitioned into training and testing sets, following a 70–30 ratio for the train-test split. The data points were standardized using the StandardScaler algorithm from the sklearn library to ensure the data compatibility with machine learning algorithms. Standardization is the process of scaling the features such that they have a mean (μ) of zero and a standard deviation (σ) of one, thereby putting them on the same scale. This is achieved by applying the following formula:


(1) $$\begin{array}{l} x^{\prime }=\frac{x-\mu }{\sigma } \end{array}$$

In Equation (1), *x*′ represents the standardized value, *x* is the original data point, μ is the mean of the feature vector, and σ is the standard deviation of the feature vector. By using this formula, we ensure that every standardized data point reflects how many standard deviations it stands from the mean of the original data. This standardization step is critical because it enables the machine learning model to converge faster and achieve higher accuracy.

**Figure 4:**
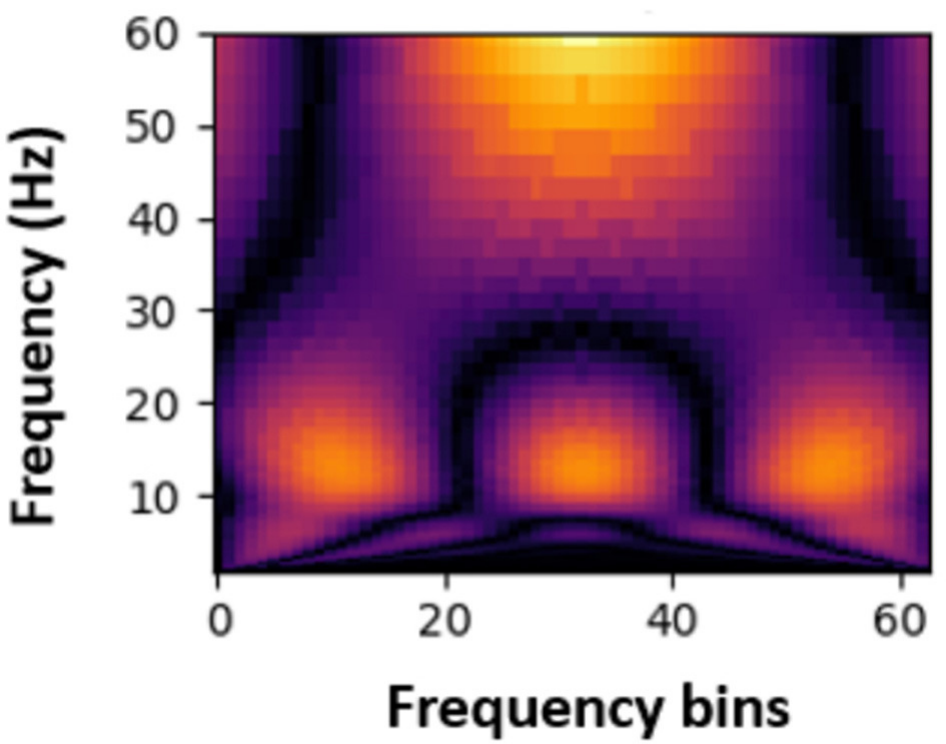
Sample spectrogram for healthy participants.

**Figure 5:**
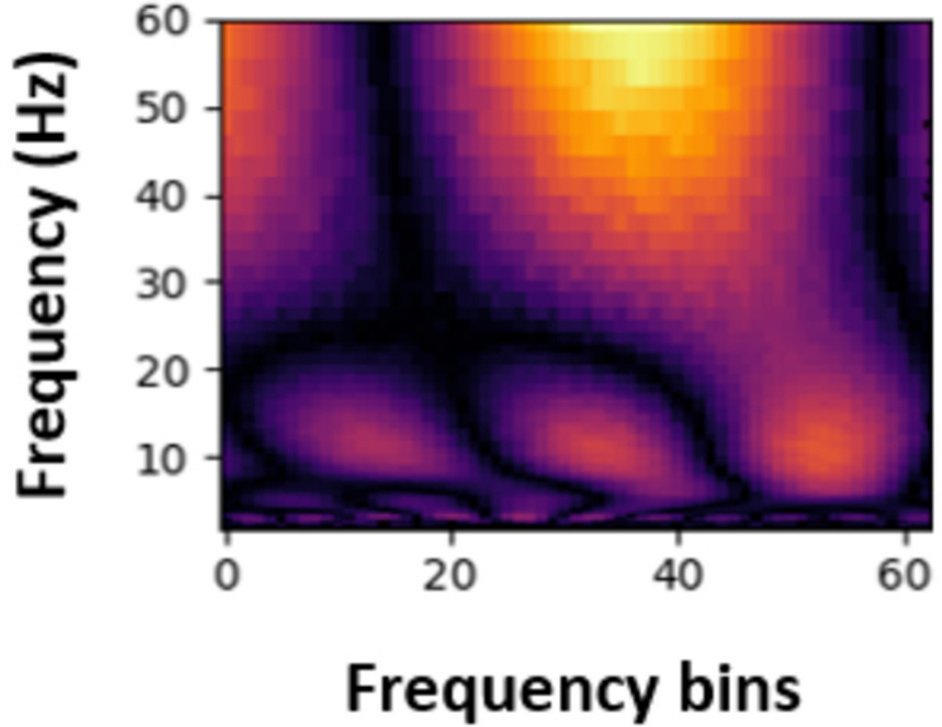
Sample spectrogram for PASC participants.

Next, the Continuous Wavelet Transform (CWT) was applied to the signals to extract time-frequency information. This transformation enabled the extraction of time and frequency features in the data, providing valuable insights for PASC and ME effects detection. The transformed data were then converted into spectrogram-like matrices (Figures 4, 5) using the CWT, which were used as input for machine learning and deep learning models. Spectrograms offer a visual representation of the frequency content of the EEG signals over time, allowing the models to learn spatial and temporal patterns effectively.

### 2.6 Synthetic data generation

#### 2.6.1 Overview

Synthetic data generation is an essential technique for augmenting datasets, especially in areas where obtaining additional real data is challenging or raises privacy issues. A similar approach has been proven successful in augmenting medical image datasets, significantly improving the classification performance (Frid-Adar et al., 2018). In this task, we create synthetic spectrogram data through Wasserstein Generative Adversarial Networks (WGANs) to discern patterns in the EEG signals of healthy individuals and those afflicted with PASC and ME. With the aid of WGANs, we generate synthetic spectrograms that are incorporated into the training phase. After the model is trained, it is then evaluated on the original, unaltered test dataset. The results, measured through evaluation metrics, highlight the benefits of integrating synthetic data into the training process. This strategy amplifies the volume of training data and addresses privacy issues since synthetic data does not directly trace back to individual participants, ensuring their anonymity.

#### 2.6.2 Generating original spectrograms

**Figure 6:**
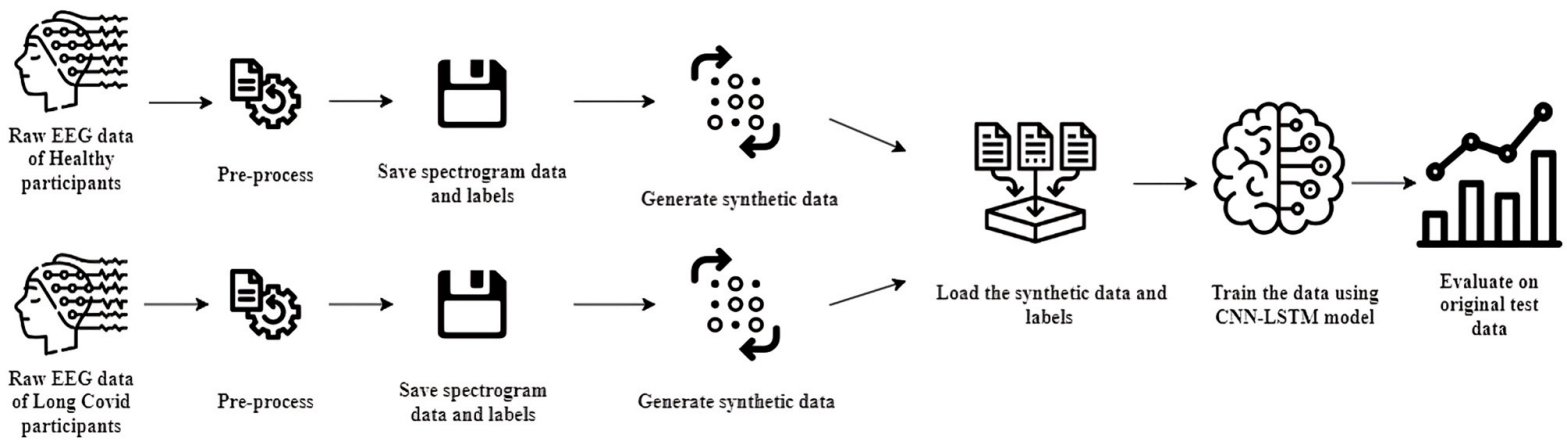
Methodology for generating synthetic spectrograms.

While our approach to generating spectrograms mirrors the original methodology outlined previously, there exists one significant distinction. In the original methodology, datasets from healthy participants and PASC/ME patients were combined and processed collectively. However, our synthetic approach processes and handles the datasets separately, as shown in Figure 6. This ensures we maintain distinct spectrogram and label data for each class, allowing for more focused analysis for both categories. Finally, each class's spectrogram and label data are saved for subsequent analyzes.

#### 2.6.3 Generating synthetic spectrograms

The process of generating synthetic spectrograms involves training a Wasserstein Generative Adversarial Network (WGAN) on the original spectrogram data to produce synthetic results. Here's a step-by-step breakdown:

- Loading data: The dataset was initially loaded from the original spectrograms, which were stored in the “.npy” format. This format is a file format used by NumPy, a library in Python for numerical computing.
- Defining the neural network architectures: The Generator is tasked with creating the synthetic data, built upon three dense layers, each supplemented with batch normalization and leaky ReLU activation functions. On the other hand, the Critic replaces the traditional discriminator in WGANs and is designed with dense layers equipped by dropout and leaky ReLU functionalities.
- Training process: Our approach used the RMSprop optimizer to train the generator and the critic. This training employed a specific loss function derived from WGANs. We initiated the training process by generating synthetic images from random inputs. Furthermore, we proceeded to train the critic and the generator sequentially. A crucial aspect of our method required maintaining the critic's weights within the range of –0.01 to 0.01, ensuring the process functioned as intended.
- Generation of synthetic data: Throughout the training cycle, every set of 100 epochs generates the synthesis of new data samples that are then appended to the synthetic dataset.
- Post-processing and saving: In this step, the synthetic data undergoes denormalization to revert to its initial scale. Subsequently, the synthetic labels are allocated, and both the data and labels are archived for further model training.

## 3 ML model development

### 3.1 Overview

This section progresses to the next stage of our study, focusing on the application of specific machine learning and deep learning models, including Support Vector Machines (SVM), Random Forest (RF), Logistic Regression (LR), Convolutional Neural Networks (CNN), and Long Short-Term Memory (LSTM) networks, to our meticulously prepared dataset. Our aim is to use these models to distinguish between healthy subjects and those diagnosed with PASC and ME, with the refined visuomotor data serving as the basis for our analysis.

### 3.2 Machine learning

Our work uses ML methodologies such as SVM, RF, and LR to categorize participants based on the features extracted from the processed EEG data. These algorithms showcase their flexibility and efficacy in various classification tasks related to PASC and ME effects detection.

#### 3.2.1 Logistic Regression

Logistic Regression (LR) is chosen for its exemplary capability in binary classification tasks, perfectly suited to differentiate between healthy individuals and those with PASC and ME within our study. The model's strength lies in its transparent relationship between predictors and the probability of outcomes, providing valuable insights into how variables affect disease risk. This transparency is particularly beneficial in a clinical research setting, where understanding variable impacts is key. Although LR assumes a linear relationship between predictors and outcomes, which might oversimplify the complex patterns in EEG data, this limitation can be addressed through sophisticated feature engineering techniques. Additionally, LR's effectiveness is bolstered by regularization techniques, like L1 and L2, which help prevent overfitting by penalizing large coefficients, thus enhancing the model's predictive performance and generalizability. The strategic selection of LR, therefore, balances its computational efficiency and ease of interpretation against its limitations, making it a robust choice for analyzing the nuanced dynamics of neurological conditions in our dataset.

#### 3.2.2 Support Vector Machines

Support Vector Machines (SVM) are selected for their exceptional efficacy in managing high-dimensional spaces, which is particularly relevant for EEG data analysis within our research. The strength of SVMs lies in their ability to form a hyperplane or multiple hyperplanes in such spaces, facilitating classification, regression, and other types of analyzes. The key advantage of using SVM lies in its capacity to handle complex data structures through the use of kernel functions, enabling it to model nonlinear relationships effectively. This capability is crucial for analyzing the nuanced patterns present in neurological conditions. However, the selection of the appropriate kernel and the tuning of hyperparameters require careful consideration to prevent model overfitting. Despite these considerations, the ability of SVMs to transform feature spaces and optimize margins renders them an invaluable asset for identifying the nuanced variances in brain activity indicative of PASC and ME. The intentional application of SVMs in our research utilizes their advanced mathematical basis to derive meaningful insights from the EEG data, highlighting their importance in the field of detailed diagnostic methods.

#### 3.2.3 Random Forest

Random Forest (RF) is chosen for its ensemble learning approach, which integrates the predictions from multiple decision trees to improve accuracy and control overfitting—common issues in machine learning models. RF's capability for both classification and regression tasks adds to its adaptability. Within our research, the technique of generating several decision trees across different segments of the dataset and employing their average to boost accuracy and reduce overfitting is especially beneficial. This approach enables the model to capture a broad spectrum of patterns in the EEG data, which might be missed by more simplistic models. One drawback of RF is its potential for high computational complexity, especially with a large number of trees or in cases of very deep trees. A limitation of RF, however, is the potential increase in computational demand, particularly when dealing with a significant number of trees or extremely complex trees. Despite this, its effectiveness in preventing overfitting and managing datasets with intricate variable relationships renders RF crucial to our study. By implementing Random Forest, we leverage the combined strength of various decision processes to deepen our insights into detecting PASC and ME, thus enhancing the accuracy and dependability of our results.

### 3.3 Deep learning

This section offers a detailed overview of the deep learning models employed in this research, encompassing Convolutional Neural Networks (CNN), Long Short-Term Memory (LSTM) networks, and Gated Recurrent Unit (GRU) networks. These models serve as the foundation for the ConvLSTM, CNN-LSTM, GRU, and BiLSTM techniques utilized in our work.

#### 3.3.1 Convolutional Neural Networks

**Figure 7:**
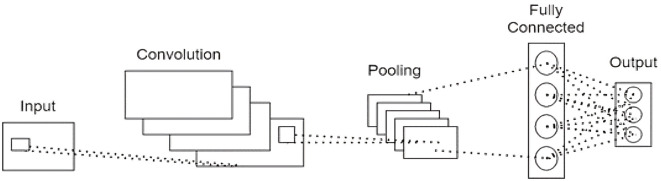
Schematic representation of a basic Convolutional Neural Network (CNN).

CNNs are a type of deep learning model designed explicitly for handling grid-like data, such as images or time series data, making them particularly suitable for our study as we have converted EEG data into spectral image-like data. Their unique structure, which typically includes convolutional layers, activation layers, pooling layers, and fully connected layers, allows them to excel at detecting patterns in such transformed data. The convolutional layers apply filters to the input data, enabling the network to learn and recognize patterns. This is especially beneficial in our context as convolutional layers are highly effective at processing image data, recognizing spatial hierarchies and patterns that traditional methods might overlook. Activation layers introduce non-linearity into the model, which allows it to learn complex patterns. Pooling layers play a critical role in reducing the spatial dimensions of the data, which helps control overfitting and minimize computation time. Lastly, fully connected layers consolidate the learned features and produce the final classification output. In this study, we have integrated CNNs into the ConvLSTM and CNN-LSTM models to efficiently extract spatial features from the transformed EEG data. For the schematic representation of the CNN architecture used in this research, refer to Figure 7.

#### 3.3.2 Long Short-Term Memory

**Figure 8:**
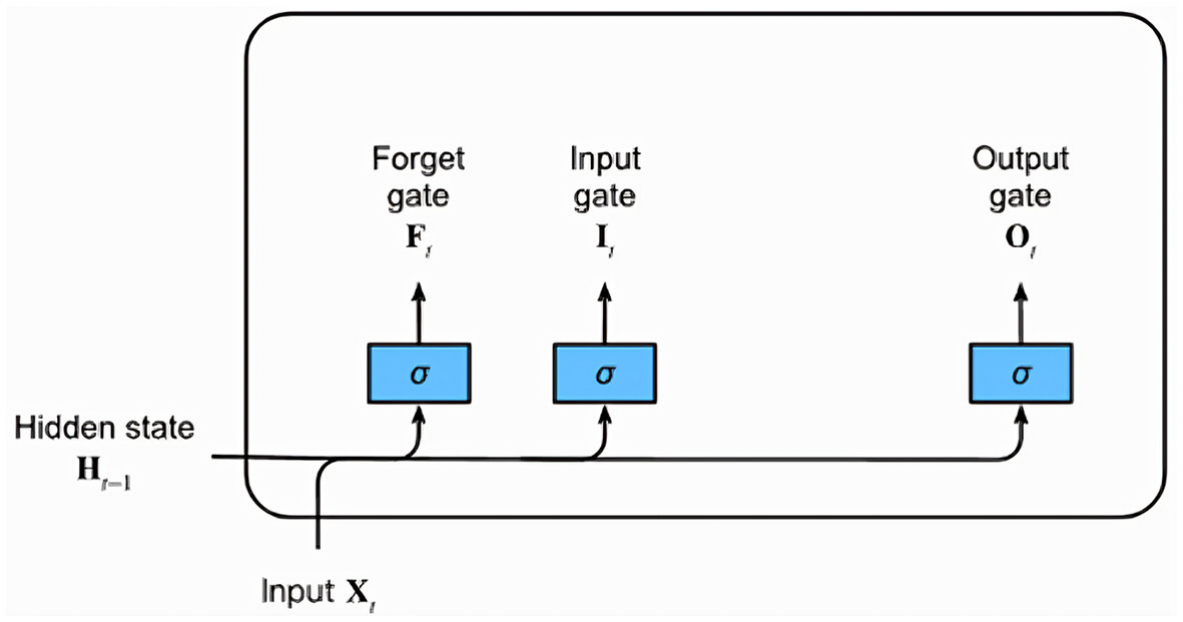
Diagram of a Long Short-Term Memory (LSTM) network highlighting the Input, Forget, and Output gates.

LSTM networks, a type of recurrent neural network (RNN) architecture, excel at learning and retaining long sequences of input data, making them particularly suited for time series and sequential data. This is largely because of their unique architecture that can capture temporal dependencies across time, crucial for the EEG data in this study. Figure 8 provides a graphical representation of a typical LSTM unit, which comprises a memory cell and three essential gates: the input, forget, and output gates.

Each gate is represented by a fully connected layer with a sigmoid activation function, resulting in output values in the range of 0–1. Intuitively, the input gate determines the extent to which the new input influences the current memory cell state. The forget gate, on the other hand, decides whether to retain or discard the current memory value. Lastly, the output gate controls the influence of the memory cell on the unit's output at the current time step. Alongside the gates, an input node computed with a *tanh* activation function is also present. This node provides a normalized version (–1 to 1) of the current input for the memory cell. The gating mechanisms combined with the memory cell enable LSTM networks to learn and recall long-term dependencies in the data, which are typically challenging for traditional RNNs. Given their ability to handle temporal patterns, this study incorporates LSTM networks into the ConvLSTM and CNN-LSTM models to efficiently learn temporal patterns from the EEG data.

#### 3.3.3 Convolutional Long Short-Term Memory

The ConvLSTM model is designed to capture both spatial and temporal dependencies in the input data, while also incorporating a self-attention mechanism. It starts with a series of convolutional layers, followed by batch normalization, activation, and dropout layers. The output of these layers is then reshaped and fed into a ConvLSTM2D layer, which combines convolutional operations with LSTM cells. After the ConvLSTM2D layer, a pooling layer is used to reduce the spatial dimensions, followed by a self-attention layer (Vaswani et al., 2017) to compute attention scores for each input data element, considering the contextual importance of each element in the input sequence. A flatten layer is used, followed by dense layers to produce the final output. This architecture enables the network to process data in a spatially localized manner while also capturing temporal patterns and considering the importance of each element in the sequence.

#### 3.3.4 CNN-LSTM

The CNN-LSTM model combines the feature extraction capabilities of Convolutional Neural Networks (CNNs) with the sequential learning ability of Long Short-Term Memory (LSTM) networks. The architecture consists of a series of convolutional layers, followed by batch normalization, activation, and dropout layers. After the CNN layers, a pooling layer is used to reduce the spatial dimensions. The output is then fed into LSTM layers to learn temporal patterns in the data. Additionally, this model incorporates a self-attention mechanism, which computes attention scores for each input data element, considering the contextual importance of each element in the input sequence.

#### 3.3.5 BiLSTM

The BiLSTM model captures information from both the past and future contexts by processing the input data in both forward and backward directions. The architecture begins with a series of convolutional layers, followed by batch normalization, activation, and dropout layers. The output from these layers is then fed into bidirectional LSTM layers, with one LSTM processing the input data in the forward direction and the other in the reverse direction. The outputs of both LSTMs are concatenated, and the combined output is fed through dense layers to produce the final output. This model is particularly effective for sequence-to-sequence learning tasks, capturing information from both directions to improve predictions.

#### 3.3.6 Gated Recurrent Unit

The GRU model is a simplified variant of the LSTM architecture, designed to capture both spatial and temporal dependencies with fewer parameters. The model starts with a series of convolutional layers, followed by batch normalization, activation, and dropout layers. The output from these layers is then fed into GRU layers, which use the update and reset gates to control the flow of information through the network. The operation of update and reset gates within the GRU model is quantitatively described by Equations 2 and 3, respectively. After the GRU layers, dense layers are used to produce the final output. This architecture achieves similar performance to LSTMs while being computationally more efficient, making it suitable for tasks involving time series analysis and natural language processing.


(2) $$\begin{array}{ll} \text{Update gate: }z_{t} & =\sigma \left(W_{z}x_{t}+U_{z}h_{t-1}+b_{z}\right) \end{array}$$

(3) $$\begin{array}{ll} \text{Reset gate: }r_{t} & =\sigma \left(W_{r}x_{t}+U_{r}h_{t-1}+b_{r}\right) \end{array}$$

Where σ is the sigmoid activation function, W, U, and b are weight matrices and bias vectors.

## 4 Evaluation and results

### 4.1 Statistical analysis of model performance

In our study, we conducted a detailed evaluation of our models using 10-fold cross-validation to ensure a thorough assessment of their performance. This was followed by a statistical comparison using the Wilcoxon signed-rank test to analyze the accuracy of four models: CNN-LSTM, ConvLSTM, BiLSTM, and GRU. The null hypothesis for our analysis was that there is no significant difference in the accuracies between any two models being compared. Our analysis focused on identifying statistically significant differences in model accuracies, with results detailed in Table 2. The analysis revealed no significant differences in performance between CNN-LSTM, ConvLSTM, and GRU, indicating that their abilities to classify EEG patterns related to PASC and ME are statistically comparable, thereby failing to reject the null hypothesis for these comparisons. However, when comparing the BiLSTM model against the others, significant differences were observed, suggesting that the BiLSTM model's performance distinctly varies from that of the CNN-LSTM, ConvLSTM, and GRU models, leading to the rejection of the null hypothesis in these instances.

**Table 2:** Wilcoxon signed-rank test results.

**Comparison**	***P*-value**	**Corrected *P*-value**	**Reject null hypothesis**
CNN-LSTM vs. ConvLSTM	0.275391	0.413086	False
CNN-LSTM vs. BiLSTM	0.001953	0.003906	True
CNN-LSTM vs. GRU	0.921875	0.921875	False
ConvLSTM vs. BiLSTM	0.001953	0.003906	True
ConvLSTM vs. GRU	0.625000	0.750000	False
BiLSTM vs. GRU	0.001953	0.003906	True

The statistical analysis conducted in our study highlights the task of EEG pattern classification for PASC and ME, where no single model uniformly outperforms others across all metrics. The distinctive statistical performance of the BiLSTM model, in comparison to the CNN-LSTM, ConvLSTM, and GRU models, underscores the variability in model efficacy under different evaluation frameworks. This variability suggests that the choice of model cannot be predicated solely on one type of performance assessment, such as accuracy from conventional train-test splits or cross-validation. It points to the necessity of a diversified evaluation strategy, considering the varied nature of EEG data and the specific requirements of real-world applications. This variability among models points to the importance of understanding each model's performance nuances, including how they handle EEG data complexities and meet the classification goals, as key considerations in model selection.

### 4.2 Dataset composition and segmentation

Prior to evaluating the performance of our models, we took several considerations into account while preparing the training and testing datasets. Initially, we collected extensive EEG data from each participant, leading to a robust set of ~350 k data points for healthy subjects and 450 k for those with PASC + ME. Through a windowing process, we distilled this information into 7,651 segments for healthy participants and 13,799 segments for the combined group of PASC + ME participants. To balance the datasets and mitigate any potential classification bias, we equalized the number of data segments for each group, resulting in a final dataset of 7,651 segments per group.

The training and testing sets were then constructed from these balanced groups at the subject level, ensuring that all EEG data from a single participant was included in only one of the sets. This approach was taken to maintain the integrity of the subject-specific EEG patterns and avoid the model learning idiosyncratic features that could lead to overfitting. As such, 70% of the segments from each group were randomly selected to form the training set, totaling 10,761 segments, while the remaining 30% were allocated to the testing set, comprising 4,541 segments. This split was deliberately chosen to evaluate the models' ability to generalize their learned patterns to an independent dataset that they had not encountered during the training phase. Details regarding the training time and parameters of the models used in our study can be found in Table 3.

**Table 3:** Comparison of model training time and training parameters.

**Model**	**Training time (minutes)**	**Training parameters**
CNN-LSTM	4	1,443,490
ConvLSTM	16	12,197,295
GRU	6	630,086
Bi-LSTM	9	660,627

### 4.3 Model evaluation

In this study, we evaluated the performance of various machine learning (ML) and deep learning (DL) models for detecting PASC and ME effects using EEG data. The evaluation metrics employed included accuracy, precision, recall, and F1-score, with macro averaging applied for precision, recall, and F1-score. The calculation of these metrics, crucial for understanding the effectiveness of the predictive models, is based on the standard formulas represented by Equations 4–7.

The formulas for Accuracy, Precision, Recall, and F1-score are as follows:

Let *TP* = True Positives, *FP* = False Positives, *TN* = True Negatives, *FN* = False Negatives.


(4) $$\begin{array}{ll} \text{Accuracy} & =\frac{TP+TN}{TP+TN+FP+FN} \end{array}$$

(5) $$\begin{array}{ll} \text{Precision} & =\frac{TP}{TP+FP} \end{array}$$

(6) $$\begin{array}{ll} \text{Recall} & =\frac{TP}{TP+FN} \end{array}$$

(7) $$\begin{array}{ll} \text{F1-score} & =2\times \frac{\text{Precision}\times \text{Recall}}{\text{Precision}+\text{Recall}} \end{array}$$

Upon conducting a comparative analysis, it was found that deep learning models outperformed their machine learning counterparts. Detailed performance metrics for each of these models are provided in Tables 4, 5. Table 4 specifically presents the metrics for the ML models. Among these models, the Random Forest model achieved the best performance with an accuracy of 77%, indicating that its ensemble learning approach provided a more reliable and stable classification model for detecting effects of PASC and ME. The Logistic Regression model attained a 63% accuracy, accompanied by average accuracy of 63% for precision, recall, and F1-score. In comparison, the Support Vector Machine model demonstrated a 51% accuracy and achieved accuracy of 55% for precision, 51% for recall, and 41% for F1-score.

**Table 4:** Performance metrics comparison for machine learning models.

**Model**	**Accuracy**	**Precision**	**Recall**	**F1-score**
LR	63%	62%	64%	63%
RF	77%	72%	75%	74%
SVM	51%	55%	51%	42%

**Table 5:** Evaluation of deep learning models based on key performance metrics.

**Model**	**Accuracy**	**Precision**	**Recall**	**F1-score**
CNN-LSTM	83%	85%	82%	83%
ConvLSTM	77%	80%	77%	77%
GRU	63%	64%	63%	62%
Bi-LSTM	70%	71%	70%	70%

As shown in Table 5, where the performance metrics for DL models are presented, ConvLSTM and CNN-LSTM models showed promising results, with the CNN-LSTM model demonstrating the highest performance, exhibiting an accuracy of 83%, precision of 85%, recall of 82%, and an F1-score of 83%.

The superior performance of the CNN-LSTM model can be attributed to its unique combination and order of CNN and LSTM layers. Unlike other models, the CNN-LSTM architecture starts with CNN layers, which are excellent at extracting spatial features from the data. It then feeds these features into LSTM layers, which excel at capturing temporal dependencies in sequences. This model capitalizes on the strengths of both components: CNN layers efficiently process spatial information, reducing the complexity of the input before it's passed to the LSTM layers, which can then focus on extracting the time-based patterns without being overwhelmed by high dimensional raw input. Therefore, the CNN-LSTM model can analyze both spatial and temporal aspects of the data more efficiently while considering the contextual importance of each input sequence element. These findings suggest that deep learning models, particularly the CNN-LSTM model, show promise as effective tools for identification and management of PASC and ME effects using EEG data, which is crucial for the neuroscience field. Timely intervention could improve patient outcomes and contribute to a better understanding of the long-term effects of COVID-19 and ME on the brain.

### 4.4 Evaluation of synthetic spectrograms

Following the application of our synthetic data generation methodology, the model underwent extensive classification metric assessments, the detailed results of which are presented in Table 6. Over the course of five separate runs, the results consistently highlighted the model's proficiency in differentiating EEG patterns between healthy participants and those affected by PASC and ME. By averaging the metrics across these runs, the model demonstrated a high level of performance, achieving ~93% in precision, recall, and F1-score for both classes. Notably, the consistent average accuracy of 93% across all runs underscores the model's robustness and the effectiveness of incorporating synthetic data in the training process.

**Table 6:** Performance metrics across multiple runs: “0” indicates healthy individuals and “1” indicates PASC and ME individuals.

**Run**	**Class**	**Precision**	**Recall**	**F1-score**	**Accuracy**
1	0	90%	97%	94%	94%
	1	97%	90%	94%	
2	0	87%	97%	92%	92%
	1	97%	87%	92%	
3	0	92%	98%	95%	95%
	1	98%	92%	95%	
4	0	92%	97%	94%	94%
	1	97%	92%	94%	
5	0	85%	97%	91%	90%
	1	97%	84%	90%	
	**Avg. accuracy**				93%

This decision to train models exclusively on synthetic inputs, rather than a combination of synthetic and original inputs, was guided by several key considerations. Our methodology aimed to evaluate the models' robustness and adaptability to data that simulates real-world variability but does not carry the same potential biases or limitations inherent to the original dataset. Training exclusively on synthetic data provided a clear baseline to assess the effectiveness of data augmentation techniques in enhancing model performance. It allowed for a straightforward comparison between models trained on purely original data versus those trained on synthetic data, thereby isolating the impact of synthetic data generation on model accuracy.

Moreover, this approach facilitated a controlled experiment to assess the synthetic data's value in training robust and generalizable models. By training the models solely on synthetic inputs, we aimed to explore the efficacy of synthetic data in compensating for the limitations of the original dataset, such as limited volume and privacy concerns.

While the current phase of our research did not explore the combined use of augmented (synthetic) and original inputs during training, we acknowledge this as an important area for future investigation. Combining both data types could potentially leverage the strengths of each—enhancing model performance further by providing a richer and more diverse training set. Future work will aim to explore this possibility, examining the optimal ratio of synthetic to original data and its effects on model efficacy and reliability in real-world applications.

### 4.5 Comparison of original vs. synthetic spectrograms

In this segment, a comparison was made between the performance metrics of the CNN-LSTM model trained on original and synthetic data. While the original data-trained CNN-LSTM achieved an accuracy of 83%, the synthetic data-trained model registered a commendable average accuracy of 93% over multiple runs. Additionally, we compared the mean and standard deviation differences between the original and synthetic datasets, as shown in Table 7. It is worth noting that the values in both datasets were normalized, accounting for the low magnitudes observed in the mean and standard deviation. The standard deviation of the original dataset is ~42.74, which closely aligns with the 44.54 observed in the synthetic dataset, with a minor difference of 1.80, reflecting a percentage difference of ~4%. On the other hand, the original dataset features a mean of ~810.74, similar to the synthetic data's mean of 851.28 with a minor difference of 40.54, which translates to a percentage difference of ~5%. These results suggest that the synthetic data generation methodology is reliable and offers an enhanced performance potential when training the model. Moreover, it indicates the potential of synthetic data to augment existing datasets and significantly enhance the predictive capabilities of deep learning models, especially in areas where the collection of extensive real-world data might be challenging or time-consuming.

**Table 7:** Comparison between original and synthetic datasets.

**Metric**	**Original dataset**	**Synthetic dataset**	**Difference**
Mean (μ*V*)	810.74	851.28	40.54
Std. dev.	42.74	44.54	1.80

## 5 Conclusion and discussion

In conclusion, our study demonstrates the potential of using EEG data combined with machine learning and deep learning techniques to detect and differentiate between healthy participants and those affected by PASC and ME. The findings suggest that deep learning models, particularly the CNN-LSTM model, are promising tools for the detection and management of PASC and ME neurological effects, as evidenced by their superior performance compared to traditional machine learning approaches such as SVM, RF, and LR. The ConvLSTM and CNN-LSTM models effectively captured spatial and temporal dependencies in the data, contributing to their remarkable performance.

A significant advancement in our research is the introduction of synthetic spectrograms generated through Wasserstein Generative Adversarial Networks (WGANs). These synthetic spectrograms were used in a separate training phase and notably achieved an average accuracy of 93%, enhancing performance metrics. This success underlines the utility of synthetic data in training models where real-world data is limited or raises privacy concerns. These insights have significant implications for the neuroscience field, where detection and intervention could lead to improved patient outcomes and a better understanding of the long-term effects of COVID-19 and ME on the brain. The study also highlights the importance of data preprocessing and feature engineering in developing high-quality datasets for training and testing machine learning and deep learning models. The utilization of Continuous Wavelet Transform and spectrogram-inspired matrices facilitated the efficient extraction of time and frequency characteristics from the EEG data, yielding crucial insights for identifying the effects of PASC and ME.

In our study, the Wilcoxon signed-rank test revealed critical insights into how well CNN-LSTM, ConvLSTM, BiLSTM, and GRU models distinguish between EEG patterns of healthy participants and those diagnosed with PASC and ME. Our statistical analysis found no significant differences in efficacy among CNN-LSTM, ConvLSTM, and GRU, suggesting these models are equally viable choices based on accuracy. This finding implies that selection among these models could instead consider factors like computational efficiency or ease of use. While differences were noted with the BiLSTM model, no single model consistently outperformed the others across all metrics, highlighting the importance of choosing a model based on the specific context and needs of the task.

Future research endeavors will extend beyond merely applying deep learning models to EEG data analysis toward unraveling the intricate mechanisms that often remain obscured within these models' operational processes. Motivated by pioneering studies such as the one introducing interpretable sinc-convolutional neural networks for EEG motor execution decoding (Borra et al., 2020), we aim to delve deeper into the interpretability of the deep learning models utilized in our investigations. This groundbreaking approach has demonstrated that it is possible to integrate a sinc-convolutional layer into a convolutional neural network, enabling a transparent interpretation of the learned filters directly in the frequency domain. Such an advancement not only allows for a slight increase in decoding accuracy but also facilitates a granular understanding of the EEG features that are most pivotal for model predictions, such as the significant role of the high Γ band and the spatial localization of EEG electrodes contributing to movement decoding.

Inspired by these findings, our future work will prioritize the exploration of similar interpretable architectures and techniques, including gradient-based analyzes, to ascertain the relevance of specific EEG bands and electrode placements in the context of PASC and ME. Furthermore, building on the work of Farahat et al. (2019), which systematically evaluated convolutional neural networks for EEG signal decoding and introduced saliency maps for EEG feature visualization, we will explore new methods for visualizing the decision-making processes of neural networks. This will not only enhance the transparency and reliability of our models but also provide insights into the neural correlates of cognitive tasks, potentially revealing new EEG features and patterns critical for understanding neurological conditions.

Ultimately, this study provides a valuable foundation for developing non-invasive, efficient, and accurate diagnostic tools to detect and manage the long-term effects of both PASC and ME on the neural control of skilled motor performance, improving patient care and contributing to a deeper understanding of this complex condition. The integration of interpretability and transparency in deep learning models represents a crucial step forward in making these advanced analytical techniques more accessible and understandable for clinical neuroscience, thereby enhancing their utility in real-world applications.

## Data availability statement

The raw data supporting the conclusions of this article will be made available by the authors, without undue reservation.

## Ethics statement

The studies involving humans were approved by York University Human Participants Review Committee. The studies were conducted in accordance with the local legislation and institutional requirements. The participants provided their written informed consent to participate in this study.

## Author contributions

HA: Data curation, Formal analysis, Investigation, Methodology, Resources, Validation, Visualization, Writing – original draft, Writing – review & editing. SB: Conceptualization, Data curation, Supervision, Writing – review & editing. HE: Data curation, Funding acquisition, Methodology, Project administration, Resources, Supervision, Writing – review & editing. LS: Data curation, Project administration, Conceptualization, Formal analysis, Funding acquisition, Methodology, Resources, Software, Supervision, Validation, Visualization, Writing – review & editing. ML: Conceptualization, Investigation, Methodology, Supervision, Validation, Writing – review & editing.
